# Identification and Validation of Immune-Related Gene Signature for Predicting Lymph Node Metastasis and Prognosis in Lung Adenocarcinoma

**DOI:** 10.3389/fmolb.2021.679031

**Published:** 2021-05-24

**Authors:** Ran Jia, Zhilin Sui, Hongdian Zhang, Zhentao Yu

**Affiliations:** ^1^ Department of Esophageal Cancer, Tianjin Medical University Cancer Institute and Hospital, Key Laboratory of Cancer Prevention and Therapy of Tianjin, Tianjin’s Clinical Research Center for Cancer, National Clinical Research Center of Cancer, Tianjin, China; ^2^ Department of Thoracic Surgery, National Cancer Center, National Clinical Research Center for Cancer, Cancer Hospital & Shenzhen Hospital, Chinese Academy of Medical Sciences and PeKing Union Medical College, Shenzhen, China

**Keywords:** lung adenocarcinoma, immune-related genes, lymph node metastasis, immune cell infiltration, immune and stromal scores, immune checkpoint genes, risk score, prognosis

## Abstract

Lung cancer is a serious malignancy, and lung adenocarcinoma (LUAD) is the most common pathological subtype. Immune-related factors play an important role in lymph node metastasis. In this study, we obtained gene expression profile data for LUAD and normal tissues from the TCGA database and analyzed their immune-related genes (IRGs), and observed that 459 IRGs were differentially expressed. Further analysis of the correlation between differentially expressed IRGs and lymph node metastasis revealed 18 lymph node metastasis-associated IRGs. In addition, we analyzed the mutations status, function and pathway enrichment of these IRGs, and regulatory networks established through TF genes. We then identified eight IRGs (IKBKB, LTBR, MIF, PPARD, PPIA, PSME3, S100A6, SEMA4B) as the best predictors by LASSO Logistic analysis and used these IRGs to construct a model to predict lymph node metastasis in patients with LUAD (AUC 0.75; 95% CI: 0.7064–0.7978), and survival analysis showed that the risk score independently affected patient survival. We validated the predictive effect of risk scores on lymph node metastasis and survival using the GEO database as a validation cohort and the results showed good agreement. In addition, the risk score was highly correlated with infiltration of immune cells (mast cells activated, macrophages M2, macrophages M0 and B cells naïve), immune and stromal scores, and immune checkpoint genes (LTBR, CD40LG, EDA2R, and TNFRSF19). We identified key IRGs associated with lymph node metastasis in LUAD and constructed a reliable risk score model, which may provide valuable biomarkers for LUAD patients and further reveal the mechanism of its occurrence.

## Background

Lung cancer is one of the most common malignancies; according to statistics, 2.2 million new cases of lung cancer and led to 1.8 million lung cancer-related deaths were reported in 2020.

Non-small cell lung cancer (NSCLC) accounts for approximately 85% of lung cancers, and adenocarcinoma is the most common pathological subtype of NSCLC, which can account for more than 50% of cases (Jamal-Hanjani et al., 2017). Despite recent advances in treatment, in particular targeted therapies and immunotherapy, the 5 year survival rate for NSCLC is still less than 20% (Herzberg et al., 2017; Sung et al., 2021). Lymph node metastasis is an important cause of this suboptimal long-term survival rate. Lymph node metastasis is common even in early-stage lung adenocarcinoma (Zhang et al., 2019). In addition, lung adenocarcinoma is more likely than squamous carcinoma to metastasize to the lymph nodes (Deng et al., 2019). Patients with lymph node metastases generally require more extensive systemic therapy; thus, it is critical to identify patients at high risk for lymph node metastasis.

Immune-related factors are of great importance in cancer lymph node metastasis (Jones et al., 2018). One of the most valuable recent advances in lung cancer research has been the introduction of immunotherapy, in particular the development of immune checkpoint inhibitors such as those targeting programmed cell death protein 1 (PD-1) and programmed cell death 1 ligand 1 (PD-L1), which have greatly advanced the therapeutic approach to lung cancer (Herbst et al., 2016). The theoretical basis for immune checkpoint inhibitors derives from intensive studies of immune system function and immunosuppressive conditions in the tumor microenvironment (TME) (Singh et al., 2020). Tumor immune-related factors have an important impact on the progression and prognosis of lung cancer.

In recent years, great progress has been made with high-throughput sequencing technologies, such as microarrays and RNA-seq, and public databases consisting of large genetic and clinical datasets, such as the TCGA and GEO, have been established. In the field of lung cancer research, many studies are conducted using data obtained from public databases to identify key genes and pathways involved in cancer development and progression by analyzing gene expression profiles, and some researchers have predicted patient prognosis by constructing predictive models (Zuo et al., 2020; Ahluwalia et al., 2021).

In this study, we investigated immune-related genes (IRGs) associated with lymph node metastasis, constructed a risk score to predict lymph node metastasis, and explored the relationship between the risk score and survival and immune cell infiltration. We hope that this study will provide some valuable genetic markers for studies related to lymph node metastasis in lung adenocarcinoma.

## Method

### Data Access

A total of three publicly available datasets were used in this study, including the RNAseq dataset of LUAD from the TCGA database and the microarray data from the GSE50081 and GSE43580 datasets from the Gene Expression Omnibus (GEO) database. We averaged the gene expression of multiple probes from the same sample in the dataset and excluded cases with clinical data that lacked pathological staging or survival follow-up data (GSE43580 had no follow-up data and was used only to verify the predictive effect on lymph node metastasis). A total of 706 samples were included in the study, of which the training set comprised 450 samples from patients with LUAD and 53 samples from normal lung tissue samples from the TCGA database, and the validation set comprised 129 samples from patients with LUAD from the GSE50081 cohort and 74 samples from patients with LUAD from the GSE43580 cohort. A total of 2498 IRGs data were obtained from the ImmPort database (Bhattacharya et al., 2018). IRGs in the database were identified as key genes involved in immune activity.

In the section where the mutation status is shown, the gene mutation data used in the study were also obtained from the TCGA database.

In addition to this, we looked up immunohistochemical (IHC) images of key genes from the HPA database (https://www.proteinatlas.org/) to verify whether the expected differences were observable at the protein level.

### Differential Analysis of Gene Expression

We used the “limma” package in R software to correct for batch effects in LUAD tissue and normal tissue data and to screen for differentially expressed genes (DEGs) by Wilcoxon test. A false discovery rate (FDR) = 0.05 and a log2 |fold change| = 2 were defined as cutoff values for DEGs. We then intersected the DEGs with the list of IRGs obtained from the ImmPort database to obtain differentially expressed IRGs.

### Analysis of IRGs Associated With Lymph Node Metastases

LUAD sample data from the TCGA database were divided into two groups according to the presence or absence of lymph node metastases, and Wilcoxon test were performed on previously obtained differentially expressed IRGs using the “limma” package in R software to explore IRGs associated with lymph node metastases. A false discovery rate (FDR) = 0.05 and a log2 |fold change| = 2 were defined as cutoff values in the Wilcoxon test.

### Gene Functional Analyses

To explore the differences in pathways and functions of IRGs associated with lymph node metastases, we performed GO annotation and KEGG pathway enrichment analysis using the clusterProfiler package in R. The “GOplot” package in R was used to plot the GO annotation and KEGG pathway analysis.

### The Transcription Factors (TFs) Regulatory Network

We downloaded the list containing information on 318 TFs from the Cistrome database (http://cistrome.org/) (Mei et al., 2017). We then screened the TFs using a false discovery rate (FDR) = 0.05 and a log2 |fold change| = 2 as the cutoff values. TFs correlated with IRGs were identified by correlation analysis (correlation coefficient = 0.4, *p* value = 0.05), and Cytoscape (v3.6.0) was applied to construct protein-protein interaction (PPI) network integration of IRGs and differentially expressed TFs.

### Lymph Node Metastasis Risk Score

The LUAD cohort from the TCGA database was used as the training set to construct the lymph node metastasis risk score. We used the least absolute shrinkage and selection operator (LASSO) logistic regression algorithm of the “glmnet” package in R software to screen for the most significant IRGs for predicting lymph node metastasis and selected candidate IRGs with penalty parameter adjustment by 10-fold cross-validation (Friedman et al., 2010). The weights of the genes in the model were identified as the regression coefficients for each gene based on the optimal lambda value. For each sample, the risk score is calculated as ∑ coefficient * gene expression value. In addition, we evaluated the ability of risk scores to discriminate lymph node metastases by plotting receiver operating characteristic (ROC) curves and calculating the area under the ROC curve (AUC) using the bootstrap resampling method.

### Survival Analysis

We combined risk scores with clinical data from the LUAD cohort from the TCGA database to analyze factors affecting patient survival using univariate Cox regression and then subjected factors that significantly affected survival to multivariate Cox regression analysis to test whether risk scores independently affected patient survival. For the above analysis, the significance cutoff values were all *p* values <0.05. ROC curves and AUC values were used to evaluate the predictive effect of the risk scores on survival. In addition, we used X-tile software to find the best cutoff values to divide the cases into two groups of patients with high- and low-risk for lymph node metastasis, and Kaplan-Meier and log-rank tests were used to calculate the difference in survival between the two groups.

### Correlation Between Immune Cell Infiltration, Immune and Stromal Scores, Immune Checkpoint Expression and the Risk Score

We used the CIBERSORT package to assess the proportion of 22 leukocyte subtypes in each sample and compared the differences in immune cells in tumor versus normal tissue using the Mann-Whitney U test (Newman et al., 2015). The ESTIMATE package was used to estimate the immune and stromal scores for each tumor sample (Yoshihara et al., 2013). In addition, we selected 60 immune checkpoint genes from the B7-CD28 and TNF families, which have important roles in immune signaling in lung cancer, and assessed their correlation with risk scores. We used Spearman correlation analysis to assess the correlation between risk scores and the above three scores in tumor tissue, and *p* < 0.05 was considered significant (Croft et al., 2013; Schildberg et al., 2016).

## Result

### IRGs Associated With Lymph Node Metastases (LM-IRGs)

**FIGURE 1:**
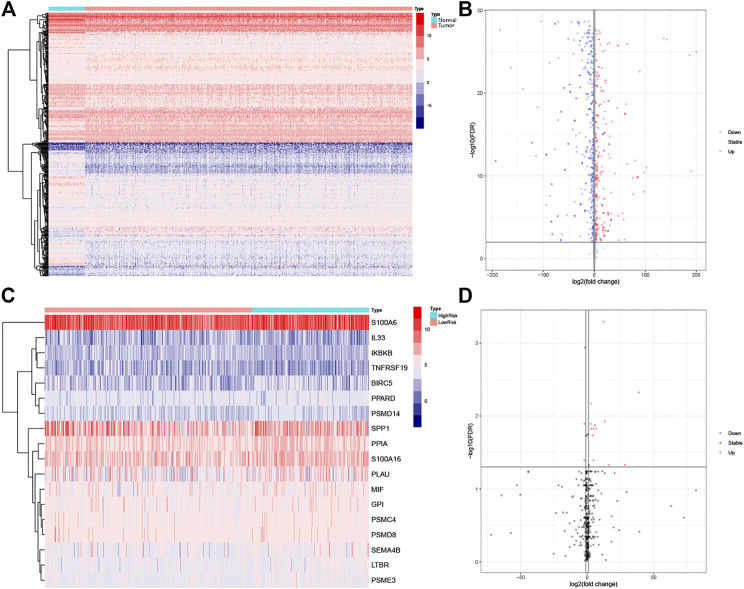
**(A)** Heatmap and **(B)** volcano plot of differentially expressed IRGs between LUAD and normal tissues. **(C)** Heatmap and **(D)** volcano plot of IRGs associated with lymph node metastases. The blue to red spectrum in **(A,C)** indicates low to high gene expression. In **(B,D)**, the blue dots represent downregulated genes, the red dots represent upregulated genes and the black dots represent genes that were not significantly differentially expressed.

A total of 6,091 differentially expressed genes were identified by differential gene expression analysis between lung adenocarcinoma tissue and matched normal controls from the TCGA database, of which the expression of 2021 was downregulated and the expression of 4,070 was upregulated. The differentially expressed gene data were then combined with data on 2,498 IRGs from ImmPort and resulted in the identification of 459 differentially expressed IRGs, of which the expression of 250 was downregulated and the expression of 209 was upregulated (Figures 1A,B). The gene matrix of the above 459 IRGs was next differentially analyzed according to the presence or absence of lymph node metastasis, and 18 LM-IRGs were identified (Figures 1C,D).

### Functional Enrichment Analysis

**FIGURE 2:**
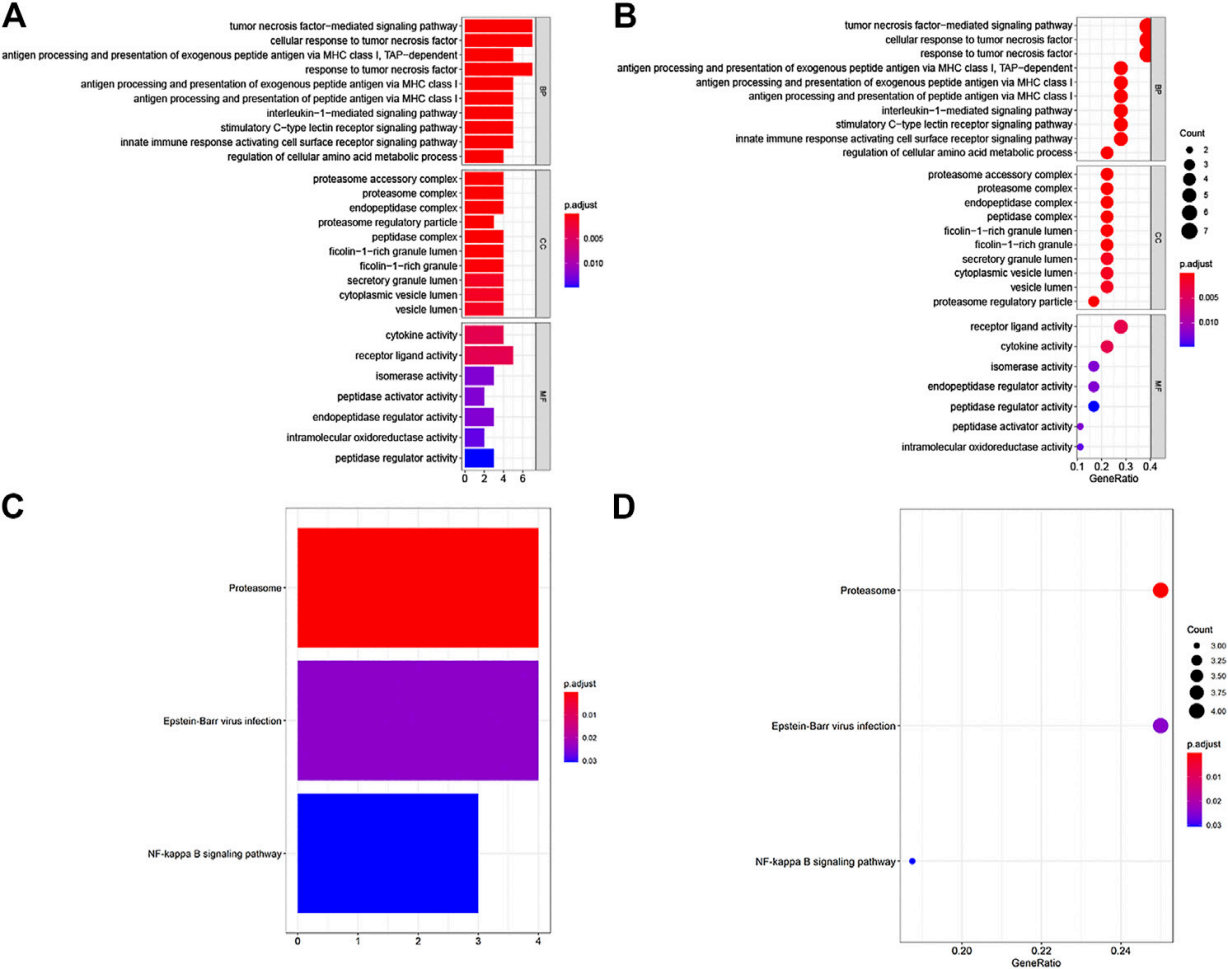
GO and KEGG enrichment analysis of IRGs associated with lymph node metastases. **(A,B)** GO analysis: BP represent biological process, CC indicated cellular component and MF represented molecular function, respectively. **(C,D)** KEGG pathways analysis.

For the LM-IRG gene signature, GO and KEGG enrichment analysis was performed using the R software “clusterProfiler” package based on expression differences between subgroups with and without lymph node metastasis. Setting *p* = 0.05 as the cutoff value for significance, we found that 190 BP, 15 CC, and 7MF were enriched in GO, and three pathways were enriched in KEGG. The top 10 items in each category are shown in Figure 2. GO enrichment was mainly identified in GO:0033209(BP): tumor necrosis factor-mediated signaling pathway, GO:0022624(CC): proteasome accessory complex, GO:0005125(MF): cytokine activity (Figures 2A,B). KEGG enrichment included “Proteasome”, “Epstein-Barr virus infection” and “NF-kappa B signaling pathway” (Figures 2C,D).

### Mutations in LM-IRGs

**FIGURE 3:**
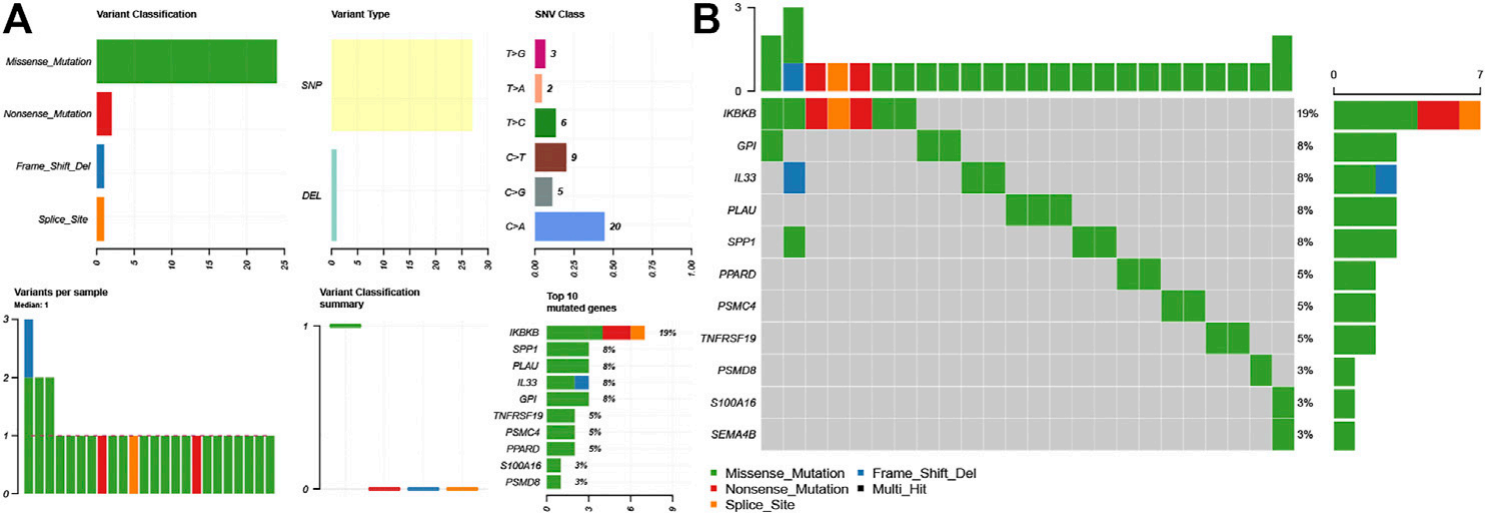
**(A)** Overview of differentially expressed IRGs mutations. **(B)** Waterfall of the mutated genes in IRGs associated with lymph node metastases.

We downloaded LUAD mutation data from the TCGA database. The findings revealed that SNPs were the most common variant type in LM-IRGs (Figure 3A). Based on the MutSigCV algorithm, the waterfall diagram showed the integration status of somatic mutations in LUAD, and the results showed that the somatic mutations rate of IKBKB was relatively high (19%), and GPI (8%),IL33 (8%), PLAU (8%), SPP1(8%) had a moderate mutation rate (Figure 3B).

### The Regulatory Network for LM-IRGs and TFs

**FIGURE 4:**
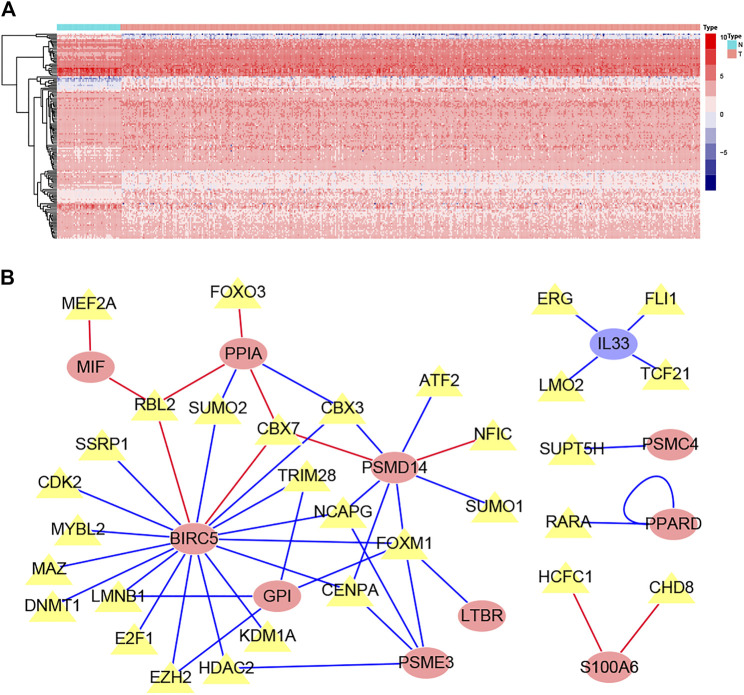
**(A)** Heatmap of differentially expressed TF genes between LUAD and normal tissues. **(B)** Regulatory network constructed based on differentially expressed TFs and IRGs associated with lymph node metastases. The yellow triangles indicated TFs, the yellow ellipse indicated IRGs that not related to survival, the red oval indicated IRGs related to poor prognosis, the blue oval indicated IRGs related to good prognosis.

The list containing 318 TF genes was obtained from the Cistrome database, and 144 differentially expressed TFs were screened from previously obtained DEGs in normal and tumor tissues. We then plotted the heatmaps of these TFs (Figure 4A). To explore the potential regulatory mechanisms of LM-IRGs, we constructed regulatory networks based on the above 144 TFs and 18 LM-IRGs. The cutoff values in the correlation analysis were correlation coefficient > 0.4, and a *p* value < 0.05. Finally, the PPI network was visualized using Cytoscape software (Figure 4B).

### Lymph Node Metastasis Risk Score and External Validation

**FIGURE 5:**
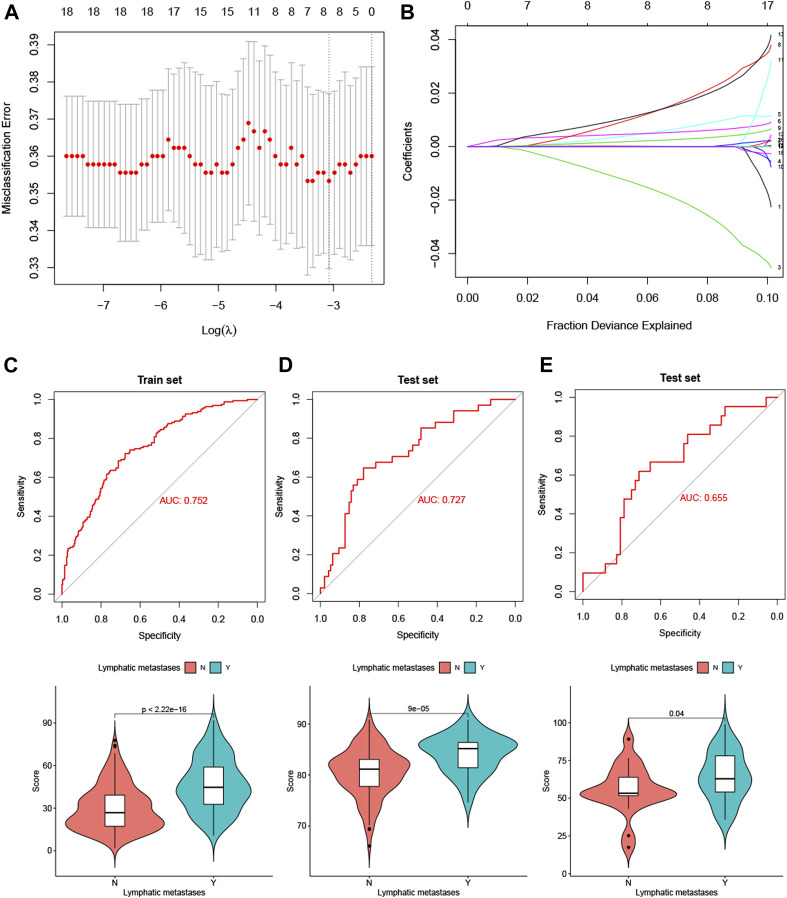
**(A)** Selection of tuning parameter (λ) in the LASSO model used 10-fold cross-validation in TCGA training set. **(B)** LASSO coefficient profiles of the 18 LM-IRGs. A coefficient profile plot was produced against the log(λ) sequence. **(C–E)** ROC curves and vioplots showing discrimination ability of the risk score in the TCGA training set **(C)**, GSE50081 validation set **(D)** and GSE 43580 validation set **(E)**.

We performed a LASSO logistic regression analysis of the gene expression matrix of 18 LM-IRGs from the LUAD cohort of the TCGA database by the presence or absence of lymph node metastases, with an optimal tuning parameter value (SE) of 0.1358 and a lambda value of 0.046, identifying the risk score constructed using the eight most relevant genes (Figure 5A,B). The formula for calculating the risk score was as follows:

Risk score =−3.25* IKBKB+ 1.05* LTBR+ 0.67* MIF+ 2.60 *PPARD+ 0.40 *PPIA+ 2.39 *PSME3 +0.0085 *S100A6 +0.074 *SEMA4B.

The risk scores were then calculatd for the training set TCGA LUAD cohort (Figure 5C) and the validation sets GSE50081 (Figure 5D) and GSE43580 (Figure 5E), and the ROC curves were plotted with AUC values of 0.75 (95% CI: 0.7064–0.7978), 0.72 (95% CI: 0.6288–0.825) and 0.65 (95% CI: 0.5179–0.7916), respectively. In addition, we also analyzed the differences in risk scores between the groups with and without lymph node metastases using t-tests in the three cohorts separately, and the results showed that the *p*-values for all three groups were <0.05 (Figures 5C–E).

### Correlation of Risk Scores With Survival and External Validation

**FIGURE 6:**
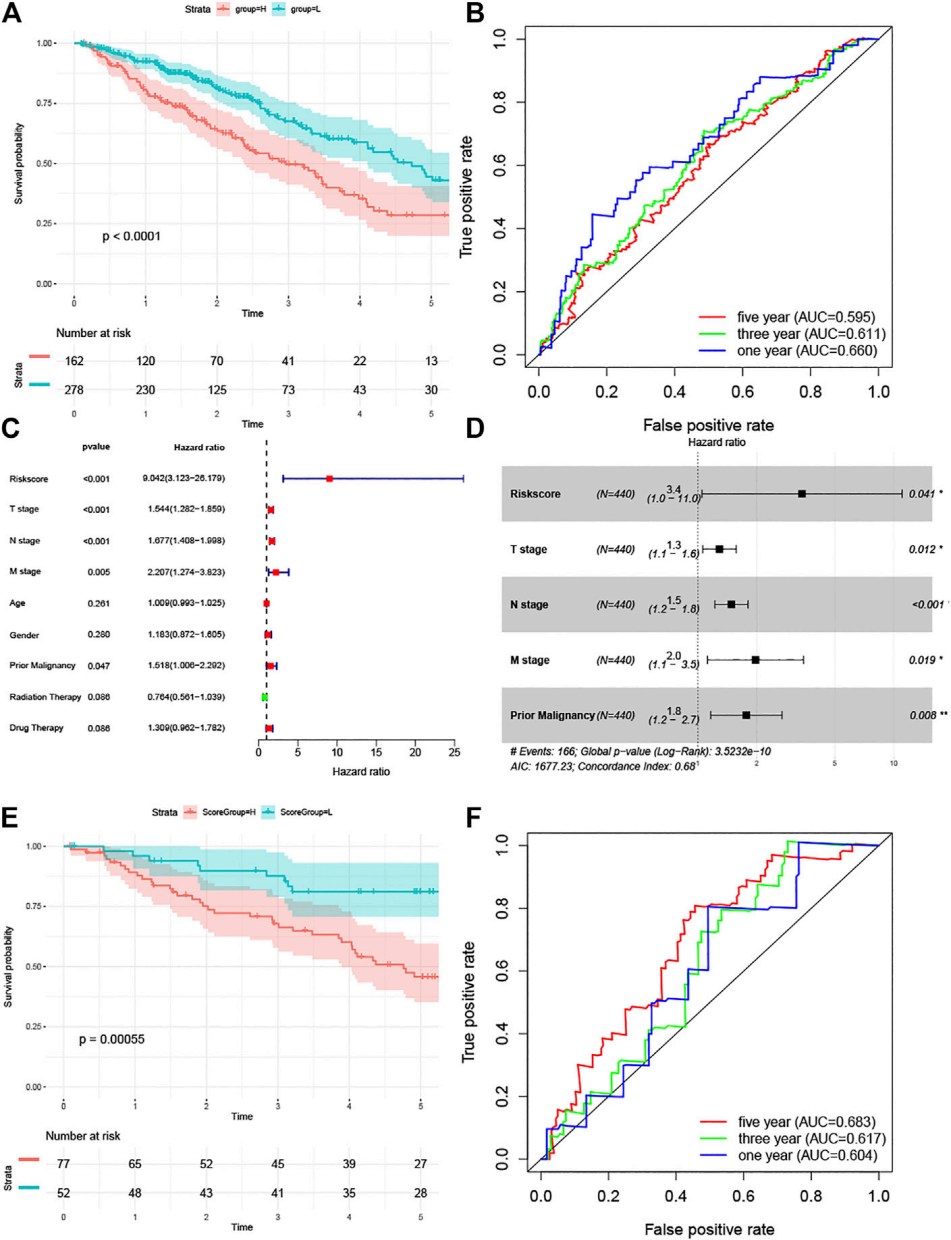
**(A)** Kaplan-Meier curve analysis of the TCGA training set. **(B)** Survival-dependent receiver operating characteristic (ROC) curve indicated prognostic results of the risks core. The area under curve (AUC) of the TCGA training set corresponding to 1, 3, and 5 years survival was provided. **(C)**The univariate Cox analysis and **(D)** multivariate Cox analysis of the TCGA training set for evaluating the prognostic value of the risk score. **(E)** Kaplan-Meier curve analysis of the GSE50081 validation set. **(F)** Survival-dependent receiver operating characteristic (ROC) curve of the GSE50081 validation set corresponding to 1,3, and 5 years survival.

Patients in the TCGA LUAD cohort were divided into high-and low-risk groups based on risk scores, and survival curves were plotted using the Kaplan-Meier method. The log-rank test showed a significant difference in survival between the two groups (Figure 6A). In addition, we also plotted survival ROC curves based on risk scores, with AUC values of 0.660, 0.611, and 0.595 for 1-, 3-, and 5-years survival, respectively (Figure 6B). To test whether the risk score independently influenced survival, we analyzed the influence of age, gender, T stage, N stage, M stage, prior malignancy, and treatment modality (radiotherapy and chemotherapy only, no surgery cases in TCGA cohort) by univariate Cox regression analysis together with the risk score, and T stage, N stage, M stage, prior malignancy, and risk score were found to have an effect on patient survival (Figure 6C). The above factors affecting survival were then subjected to multivariate Cox regression analysis, and as shown in Figure 6D, all five were found to independently affect survival; in particular, the risk score showed the highest hazard ratio (3.4; 95% CI 1.0–11.0). For external validation, we show in Supplementary Table S1 the baseline information for the three groups of patients. We only used the GSE50081 cohort due to the absence of survival information in the GSE43580 data, and the Kaplan-Meier survival curves showed differences in survival between patients in the high-and low-risk groups (Figure 6E), with AUC values of 0.604,0.617, and 0.683 for the survival ROC curves at 1,3, and 5 years, respectively (Figure 6F).

### Validation of Gene Signature at the Protein Level

**FIGURE 7:**
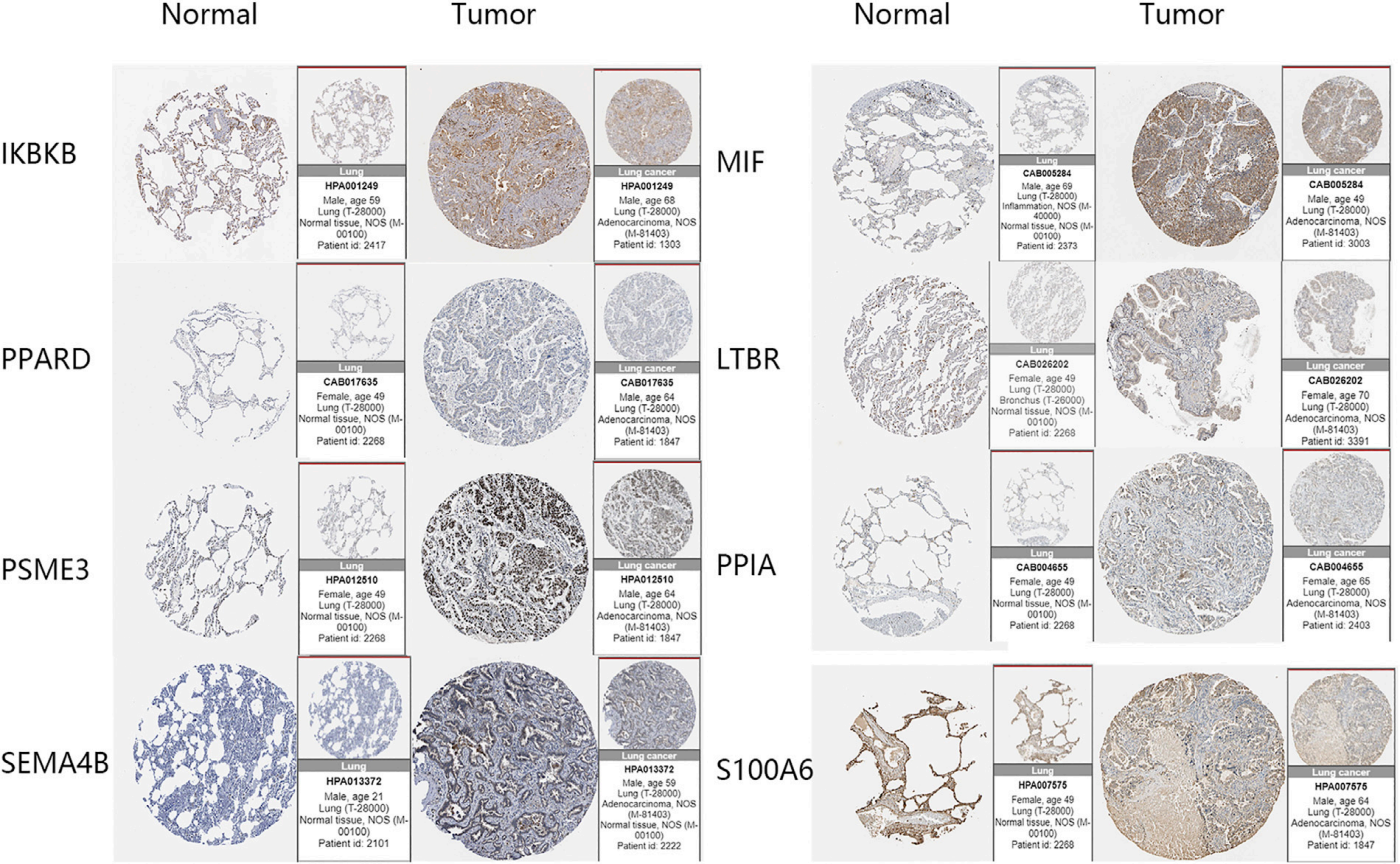
IHC images of the eight key genes in LUAD and normal tissues obtained from the HPA database.

Immunohistochemical data from the HPA database were used to validate the risk score-related LM-IRGs. The results showed that IKBKB, LTBR, MIF, PPIA, PSME3, and SEMA4B were all more highly expressed in the tumor, as we expected, but PPARD staining was not detected in either tumor or normal tissues, and S100A6 was expressed in both normal and tumors and the expression level did not appear to be significantly different (Figure 7).

### Correlation Between Risk Score and Immune Cell Infiltration

**FIGURE 8:**
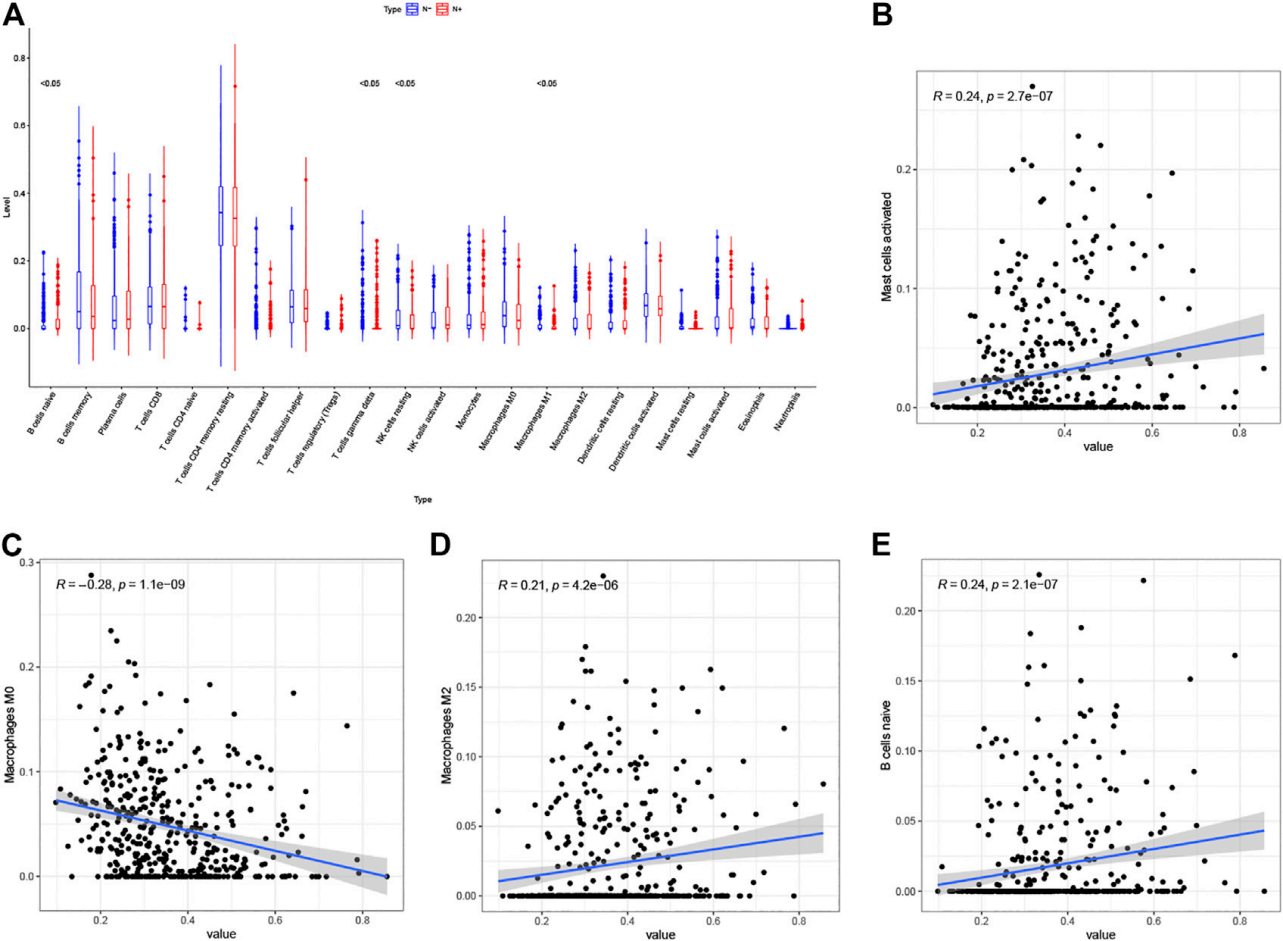
**(A)** Differential infiltration of 22 immune cell types in LUAD with and without lymph node metastasis (Red: N+, Blue: N−). **(B–E)** Correlation analysis of risk scores with mast cells activated **(B)**, macrophages M0 **(C)**, macrophages M2 **(D)** and **(B)** cells naïve **(E)**.

We calculated the proportions of 22 immune cells in each sample by the CIBERSORT algorithm and compared the differences between tissues with and without lymph node metastasis. (Figure 8A). We then calculated correlation coefficients between the risk scores and immune cells proportions, with correlation coefficients > 0.2 and *p* < 0.05 being used as cutoff values, and found that risk scores correlated with mast cells activated, macrophages M2, macrophages M0 and B cells naïve (Figures 8B–E).

### Correlation Between Risk Score and TME Score

**FIGURE 9:**
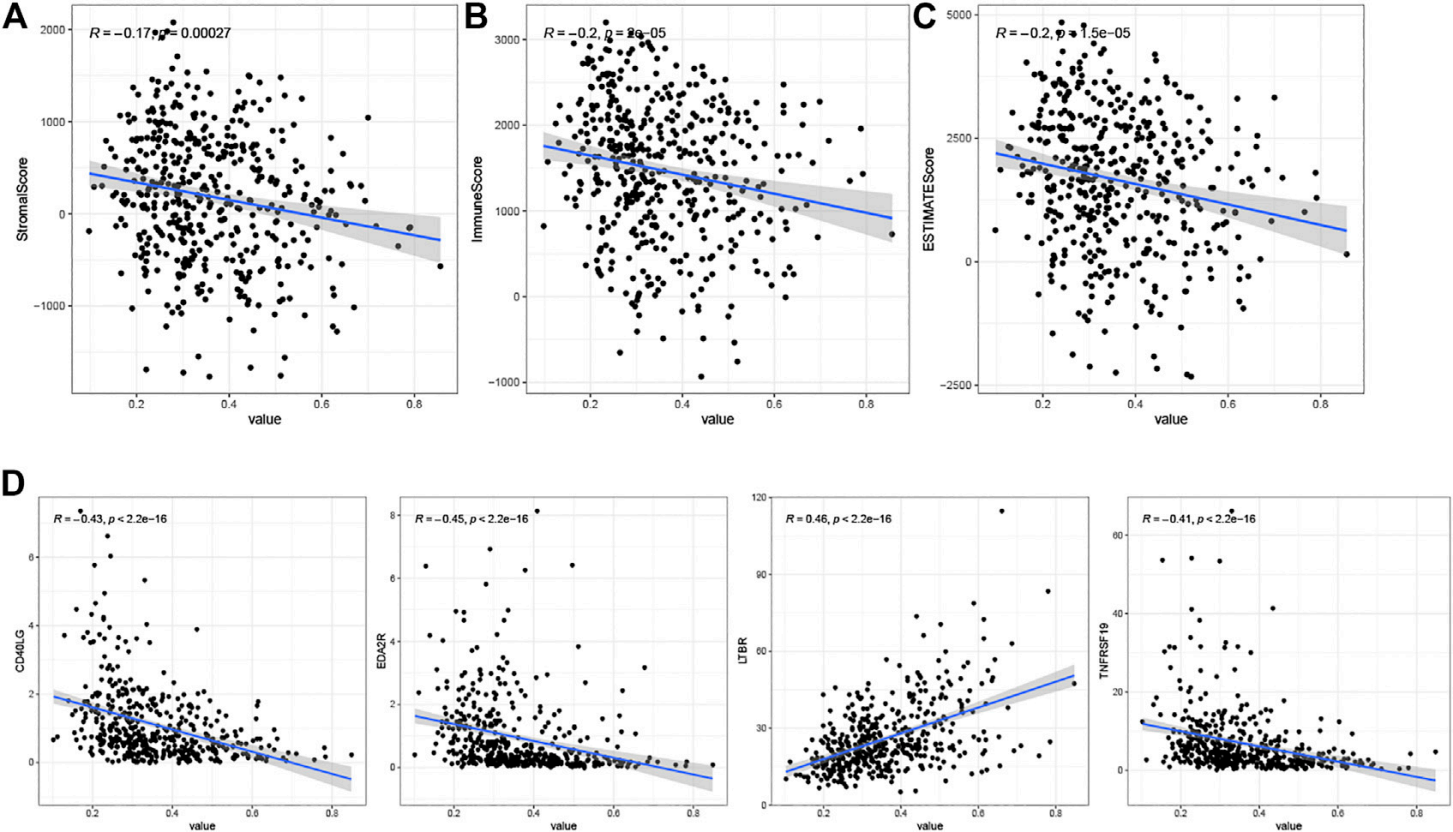
**(A)** Correlation analysis of risk scores with StromalScore. **(B)** Correlation analysis of risk scores with ImmuneScore. **(C)** Correlation analysis of risk scores with ESTIMATEScore. **(D)** Results of correlation analysis between risk scores and immune checkpoint genes.

We estimated immune and stromal scores for tumor samples using the ESTIMATE algorithm and calculated correlations with the risk score. The results showed that the risk score was positively correlated with the stromal score (Figure 9A), immune score (Figure 9B) and ESTIMATE score (Figure 9C).

### Correlation Analysis of Immune Checkpoints and Risk Score

We calculated correlation coefficients for risk scores and 60 immune checkpoint genes from the B7-CD28 and TNF families (Supplementary Table S2). To show only checkpoint genes with strong correlation with risk scores, we used correlation coefficients > 0.4 and *p* < 0.05 as cutoff values. The results showed positive correlations between risk scores and the immune checkpoint gene LTBR, and negative correlations with CD40LG, EDA2R, and TNFRSF19 (Figure 9D).

## Discussion

Lung cancer is the leading cause of death among all malignancies each year, and lung adenocarcinoma is the most common pathological subtype of lung cancer. Lung adenocarcinoma is prone to early lymph node metastasis. We reviewed the SEER database (Myers and Ries, 1989) for cases with definite 7th edition AJCC pathological stages between 2010 and 2015, and found that even in patients with T1 stage, 54.2% (4,907/9,049) had lymph node metastasis, while the proportion of lymph node metastasis did not increase significantly with the increase of T stage [T2: 49.9% (5,382/10,793), T3: 65.2% (6,245/9,582), T4: 73.1% (8,991/12,296)]. Clarification of lymph node metastasis status is crucial for the treatment of patients as well as for the assessment of their prognosis (Hwang et al., 2020). The commonly used noninvasive clinical examinations are CT and PET/CT, but these two modalities are not accurate enough for the diagnosis of lymph node metastasis and there is a certain possibility of missing the diagnosis (Li et al., 2013; El-Sherief et al., 2017). EBUS-TBNA is a valuable technique, with a higher diagnostic accuracy than PET or CT, but its use is limited to the peri-airway area and transbronchial needle aspiration is associated with some risks(Souma et al., 2020). Therefore, the development of new predictors of lymph node metastasis is of value for diagnosis.

According to previous studies on lymph node metastasis of lung cancer, many immune-related factorsplay an important role. Given the complex role of immune-related factors in lymph node metastasis, we aimed to predict the risk of lymph node metastasis using RNA sequencing data from a large number of IRGs obtained by high-throughput sequencing technology. By differential expression analysis and LASSO logistic regression analysis we constructed an 8-gene (IKBKB, LTBR, MIF, PPARD, PPIA, PSME3, S100A6, SEMA4B) based risk score model to predict lymph node metastasis in lung adenocarcinoma. IKBKB/IKKβ, the core catalytic subunit of the κB kinase complex, is involved in mediating the classical NF-κB pathway, which plays an important role in tumor initiation by inducing DNA damage, oncogenic mutation and genomic instability. In addition, NF-κB enhances tumor cell proliferation by promoting the production of multiple cytokines, growth factors and cell cycle proteins (Park and Hong, 2016). Previous studies have found an association between IKBKB and the prognosis of a variety of tumors, including osteosarcoma, gastric cancer, skin cancer, and breast cancer (Gong et al., 2020; Pan et al., 2020). Lymphotoxin β receptor (LTBR), a member of the tumor necrosis factor (TNF)/TNF receptor superfamily, significantly affects the activation and clonal expansion of CD8^+^ T cells and has been found to be associated with the development and progression of hepatocellular carcinoma (Zhu et al., 2017). Macrophage migration inhibitory factor (MIF) was originally found to act as a proinflammatory cytokine in immune and inflammatory responses, to mediate the regulation of macrophage function to counteract the anti-inflammatory activity of glucocorticoids, and to have tumor-promoting properties (Conroy et al., 2010). MIF has been extensively studied in relation to malignancy and its effects are thought to occur mainly through alteration of the tumor microenvironment (Mitchell and Yaddanapudi, 2014). Peroxisome proliferator-activated receptor-δ (PPARD) is a nuclear transcription receptor that, once activated by a ligand, binds to the promoter of a target gene and is involved in the regulation of many molecular processes (Xu et al., 2013). PPARD expression is upregulated in many malignancies, and its role in tumorigenesis remains controversial, but recent studies have revealed it its important role in metastasis and corroborate our findings (Zuo et al., 2017). Peptidyl prolyl isomerase A (PPIA) catalyzes the cis-trans isomerization of prolyl acyl peptide bonds in oligopeptides and accelerates protein folding and may play a role in cyclosporin A-mediated immunosuppression. Previous studies have revealed a correlation between PPIA and the prognosis of non-small cell lung cancer (Campa et al., 2003). Proteasome activator subunit 3 (PSME3), a subunit of the 11SREG-γ proteasome regulator, is associated with the proteasome and is known to regulate the degradation of the cell cycle protein-dependent kinase inhibitors p21 and p16, the oncogene SRC-3 and the tumor suppressor p53 (Li et al., 2007; Zhang and Zhang, 2008). In addition, recent studies have revealed that tumors evade immune surveillance by overexpressing PSME3 (Boulpicante et al., 2020). It was proposed in previous studies that S100A6 acts as a calcium sensor and regulator to promote cellular calcium signaling and, in lung cancer, promotes cancer cell proliferation, invasion, and migration through P53 acetylation (Emberley et al., 2004). SEMA4B is a member of the semaphorin protein family, which primarily regulates cell migration. SEMA4B has been shown to inhibit the invasion of non-small cell lung cancer through the PI3K signaling pathway (Jian et al., 2015). However, this is contrary to our findings as we observed the gene to be highly expressed in lung adenocarcinoma at both the transcriptome and protein levels. In addition, a query of the human protein atlas database revealed that the gene was associated with poor prognosis in lung cancer; thus, we suggest that further experiments might be required to verify the role of SEMA4B.

To verify the robustness of the risk scores, we used two external validation cohorts from the GEO database, and the risk scores were valid in both cohorts. Interestingly, although the risk score was designed to predict lymph node metastasis, by survival analysis we found it to be an independent influencer of patient survival. This may be because the biological processes the model genes are involved in make it easier for the tumor cells to disseminate, and the finding also illustrates the validity of the model. Most of the previous studies on IRGs for lung cancer were conducted by constructing risk scores to predict patient survival (Chen et al., 2020b; Fu et al., 2020; Li et al., 2020), and we believe that among the factors affecting patient survival, excluding genetic markers, clinical factors such as pathological stage and treatment modality also have a great influence, and the influence of these factors is difficult to completely eliminate. However, in our study, the focus was on the effect of genetic markers on lymph node metastasis, and the biological behavior of the tumor was almost independent of the clinical factors; thus, our findings may be more accurate and more reproducible.

Immune checkpoint inhibitors have been the biggest advance in lung cancer treatment in recent years, and there is abundant evidence from basic research that an immunosuppressive TME depletes T cells and renders them unresponsive (Hanahan and Weinberg, 2011), which allows tumor cells to evade immune surveillance and clearance. Immune evasion is an important part of the lymph node metastasis mechanism (Chen et al., 2020b; Fu et al., 2020; Li et al., 2020), so we also investigated the relationship between risk scores and immune cells, immune scores and immune checkpoint genes. We calculated the proportion of stromal and immune cells in the tumor tissue using the ESTIMATE algorithm (Chen et al., 2020b; Fu et al., 2020; Li et al., 2020). The stromal and immune scores are measures of the proportion of stromal and immune cells in the tumor tissue, respectively, and the ESTIMATE score indicate tumor purity, which is defined as the percentage of tumor cells in the TME and is closely related to the prognosis of cancer. The findings showed a significant correlation between risk scores and all three of these other factors, possibly predicting an effect of the TME on lymph node metastasis (Qi et al., 2020). Tumor-infiltrating B cells are a key component of adaptive immunity, and data on the antitumor effects of B cells in NSCLC are inconsistent (Hu et al., 2019). Recent studies have revealed that naive B cells can inhibit cancer cell proliferation and are associated with a favorable prognosis in NSCLC (Chen et al., 2020a). Our results suggest that naive B cells may also have an inhibitory effect on lymph node metastasis. In addition, in the differential analysis of immune cell infiltration in patients with and without lymph node metastasis we found a higher proportion of γδ T cells in patients with lymph node metastasis. γδ T cells can secrete cytokines and exert potent cytotoxicity against a wide range of cancer cells, making them potential effector cells for cancer immunotherapy (Kakimi et al., 2014). In an *in vitro* study, γδ T cells were found to kill the N592 lung cancer cell line (Ferrarini et al., 1996). In addition, one clinical trial using γδ T cells to treat NSCLC showed that the cells had some effect (Nakajima et al., 2010). This seems to contradict our results. However, recent studies have found that microbiota contribute to inflammation and cancer progression by stimulating γδ T cells in the lung, leading to γδ T cell expansion and phenotypic changes (Jin et al., 2019). Overall, γδ T cells play a complex role in tumor immunity in lung cancer, and in our study we found higher levels of γδ T cell infiltration in patients with lymph node metastasis, the mechanism of which deserves further investigation. Costimulatory molecules expressed in cancer cells, especially immune checkpoints, play a crucial role in regulating antitumor immune responses (Turley et al., 2015), and the results in this study show that multiple members of the B7-CD28 and TNF families are associated with risk scores, which may indicate a potential role they play in lymph node metastasis. Overall, we constructed a risk score to predict lymph node metastasis and showed moderate predictive validity in the training cohort and a validation cohort. In addition, we explored the mutational and regulatory network of metastasis-related genes and investigated the correlation between the risk score and some key factors in the TME, all of which may provide some ideas for subsequent studies. However, our research also has some limitations. First, although we used other cohorts for validation, this is still a retrospective study and further prospective cohorts are needed to verify its validity. Second, it is IHC that has better utility for clinical work, and the reliability of our model has not been able to be validated in protein expression levels. Therefore, we will develop new cohorts in the next study to investigate the correlation between gene expression and protein levels in IHC. In addition, we will further investigate the relationship between lymph node metastasis and the biological functions of genes in the model and TME, etc. through experiments based on the directions demonstrated in the study. Third, due to the lack of diagnostic imaging information in the dataset used in the study, it was not possible to explore the improvement of the risk score on the accuracy of diagnostic imaging, and we will develop a new cohort to combine the risk score with diagnostic imaging in the next step to explore the improvement of the accuracy of the diagnosis of lymph node metastasis.

## Conclusion

We investigated the IRGs associated with lymph node metastasis in LUAD, and further explored the above IRGs in terms of gene regulation, gene function, gene pathway involvement and mutation status. The constructed risk score was effective in predicting not only lymph node metastasis but also patient survival, and we validated it using validation cohorts and IHC. In addition, the score has a significant correlation with immune cell infiltration, immune score, and immune checkpoint genes, which can provide some insight into the mechanisms of and therapeutic targets for LUAD.

## Data Availability

Publicly available datasets were analyzed in this study. This data can be found here: Expression profile can be accessed in The Cancer Genome Atlas (TCGA) (https://portal.gdc.cancer.gov/) and gene expression omnibus (GEO) database (https://www.ncbi.nlm.nih.gov/geo/), while immune related gene list can be retrieved from the Immunology database and Analysis Portal (IMMPORT) website (https://www.immport.org). Transcription factors (TFs) associated with cancer data can be obtained from the Cistrome project (http://www.cistrome.org/). IHC data can be obtained from the HPA database (https://www.proteinatlas.org/).
