# Supplementary material for: Identification and Validation of Immune-Related Gene Signature for Predicting Lymph Node Metastasis and Prognosis in Lung Adenocarcinoma
Source: Front Mol Biosci. 2021 May 24;8:679031. doi: 10.3389/fmolb.2021.679031 (PMC8182055; doi:10.3389/fmolb.2021.679031)
Supplement: Supplementary file 1 [file Table1.docx]

|  |  | TCGA |  | GSE50081 |  | GSE43580 |  |
| --- | --- | --- | --- | --- | --- | --- | --- |
|  |  | Number | Percentage | Number | Percentage | Number | Percentage |
| Age | Average | 64.8 |  | 68.7 |  | 63.2 |  |
| Sex | Female | 242 | 55% | 62 | 49% | 25 | 32% |
|  | Male | 218 | 45% | 65 | 51% | 52 | 68% |
| Pathological Stage | I | 240 | 55% | 92 | 72% | 41 | 53% |
|  | II | 105 | 24% | 37 | 28% | 36 | 47% |
|  | III | 74 | 17% | 0 | 0% | 0 | 0% |
|  | IV | 21 | 5% | 0 | 0% | 0 | 0% |
| N Stage | N0 | 291 | 66% | 94 | 74% | 56 | 73% |
|  | Not N0 | 149 | 34% | 35 | 26% | 21 | 27% |

Supplementary Table1. Baseline information of the three groups of patients
