# Supplementary material for: Identification and Validation of Immune-Related Gene Signature for Predicting Lymph Node Metastasis and Prognosis in Lung Adenocarcinoma
Source: Front Mol Biosci. 2021 May 24;8:679031. doi: 10.3389/fmolb.2021.679031 (PMC8182055; doi:10.3389/fmolb.2021.679031)
Supplement: Supplementary file 2 [file Table2.docx]

| Gene | Aliases | Family | cor | P value |
| --- | --- | --- | --- | --- |
| CD274 | PD-L1, B7-H1 | B7 | 0.082029 | 0.085678 |
| CD276 | B7-H3 | B7 | 0.382637 | 8.63E-17 |
| CD80 | B7-1, CD28LG1 | B7 | -0.18285 | 0.000115 |
| CD86 | B7-2, CD28LG2 | B7 | -0.1173 | 0.013817 |
| HHLA2 | B7-H5 | B7 | -0.05023 | 0.293126 |
| ICOSLG | B7-H2, CD275 | B7 | -0.07691 | 0.107153 |
| PDCD1LG2 | PD-L2, B7DC, CD273 | B7 | -0.03023 | 0.527028 |
| VTCN1 | B7-H4 | B7 | 0.04813 | 0.313794 |
| CD28 | Tp44 | CD28 | -0.29725 | 2E-10 |
| CTLA4 | CD152 | CD28 | -0.16476 | 0.000521 |
| ICOS | CD278, CVID1 | CD28 | -0.17409 | 0.000243 |
| PDCD1 | PD-1, CD279 | CD28 | 0.036179 | 0.449064 |
| TMIGD2 | CD28H | CD28 | -0.05502 | 0.249424 |
| CD27 | TNFRSF7 | TNFRSF | -0.2154 | 5.14E-06 |
| CD40 | TNFRSF5 | TNFRSF | -0.03851 | 0.42041 |
| EDA2R | TNFRSF27, XEDAR | TNFRSF | -0.45237 | 1.4E-23 |
| EDAR | EDA-A1R | TNFRSF | -0.21812 | 3.87E-06 |
| FAS | TNFRSF6, CD95 | TNFRSF | -0.02328 | 0.626186 |
| LTBR | TNFRSF3 | TNFRSF | 0.459693 | 2.17E-24 |
| NGFR | TNFRSF16, CD271 | TNFRSF | -0.30022 | 1.29E-10 |
| RELT | TNFRSF19L | TNFRSF | 0.026452 | 0.580001 |
| TNFRSF10A | TRAILR1, CD261 | TNFRSF | 0.034059 | 0.476096 |
| TNFRSF10B | TRAILR2, CD262 | TNFRSF | 0.077848 | 0.102934 |
| TNFRSF10C | TRAILR3, CD263 | TNFRSF | -0.167 | 0.000435 |
| TNFRSF10D | TRAILR4, CD264 | TNFRSF | -0.09949 | 0.036969 |
| TNFRSF11A | RANK, CD265 | TNFRSF | -0.01007 | 0.833194 |
| TNFRSF11B | OPG | TNFRSF | -0.01363 | 0.775544 |
| TNFRSF12A | FN14, TWEAKR, CD266 | TNFRSF | 0.359285 | 7.48E-15 |
| TNFRSF13B | TACI, TNFRSF14B, CD267 | TNFRSF | -0.31736 | 9.42E-12 |
| TNFRSF13C | BAFFR, CD268 | TNFRSF | -0.16843 | 0.000388 |
| TNFRSF14 | LIGHTR, HVEM, CD270 | TNFRSF | -0.16602 | 0.000471 |
| TNFRSF17 | BCMA, TNFRSF13A, CD269 | TNFRSF | -0.24058 | 3.27E-07 |
| TNFRSF18 | GITR, AITR, CD357 | TNFRSF | 0.134071 | 0.004847 |
| TNFRSF19 | TROY, TAJ | TNFRSF | -0.41191 | 1.89E-19 |
| TNFRSF1A | TNFR1, CD120A | TNFRSF | 0.363252 | 3.59E-15 |
| TNFRSF1B | TNFR2, CD120B | TNFRSF | -0.17999 | 0.000147 |
| TNFRSF21 | DR6, CD358 | TNFRSF | 0.250923 | 9.61E-08 |
| TNFRSF25 | DR3, TNFRSF12 | TNFRSF | -0.12374 | 0.009373 |
| TNFRSF4 | OX40, CD134 | TNFRSF | 0.032887 | 0.491408 |
| TNFRSF6B | DCR3 | TNFRSF | NA | NA |
| TNFRSF8 | CD30 | TNFRSF | -0.058 | 0.224659 |
| TNFRSF9 | 4-1BB, CD137, ILA | TNFRSF | 0.0532 | 0.265475 |
| CD40LG | TNFSF5, CD154, CD40L | TNFSF | -0.4304 | 2.87E-21 |
| CD70 | TNFSF7, CD27L | TNFSF | 0.057755 | 0.226648 |
| EDA | EDA-A1, EDA-A2 | TNFSF | -0.32185 | 4.62E-12 |
| FASLG | TNFSF6, CD95-L | TNFSF | -0.06043 | 0.205813 |
| LTA | TNFSF1 | TNFSF | -0.17445 | 0.000236 |
| LTB | TNFSF3 | TNFSF | -0.20452 | 1.53E-05 |
| TNF | TNFSF2, TNFA | TNFSF | -0.1571 | 0.000945 |
| TNFSF10 | TRAIL, CD253 | TNFSF | -0.19608 | 3.45E-05 |
| TNFSF11 | RANKL, CD254 | TNFSF | 0.040825 | 0.392953 |
| TNFSF12 | TWEAK | TNFSF | -0.14781 | 0.001879 |
| TNFSF13 | APRIL, CD256 | TNFSF | -0.24626 | 1.68E-07 |
| TNFSF13B | BAFF, CD257 | TNFSF | -0.09256 | 0.052358 |
| TNFSF14 | LIGHT, HVEML, CD258 | TNFSF | -0.25405 | 6.57E-08 |
| TNFSF15 | TL1A | TNFSF | -0.30645 | 5.08E-11 |
| TNFSF18 | GITRL | TNFSF | -0.18069 | 0.000138 |
| TNFSF4 | OX-40L, CD134L, CD252 | TNFSF | 0.055473 | 0.245565 |
| TNFSF8 | CD30L, CD153 | TNFSF | -0.29569 | 2.5E-10 |
| TNFSF9 | 4-1BB-L, CD137L | TNFSF | 0.210749 | 8.26E-06 |

Supplementary Table2. Checkpoint genes included in the study and the correlation coefficients and p-values of the correlation analysis
